# Not getting the nod: The case against Yao syndrome

**DOI:** 10.70962/jhi.20250224

**Published:** 2026-04-03

**Authors:** Paul E. Gray, Seth L. Masters, Edwin P. Kirk

**Affiliations:** 1 Department of Immunology and Infectious Diseases, Sydney Children’s Hospital, Randwick, Australia; 2 University of Western Sydney, Sydney, Australia; 3 Centre for Innate Immunity and Infectious Diseases, Hudson Institute of Medical Research, Clayton, Australia; 4 Department of Molecular and Translational Science, Monash University, Clayton, Australia; 5 School of Clinical Medicine, University of New South Wales, Sydney, Australia; 6 Centre for Clinical Genetics, Sydney Children’s Hospitals Network, Sydney, Australia; 7 NSW Health Pathology Randwick Genomics Laboratory, Randwick, Australia

## Abstract

NOD2-associated autoinflammatory syndrome or Yao syndrome was first described in 2011 in seven patients who were heterozygous for the *NOD2*:c.2717+158C>T (previously IVS8+158) variant, several of whom were also heterozygous for a second variant, NOD2:c.2023C>T;p.Arg675Trp (previously R702W). Since then, over 30 papers have been published either about or including mention of Yao syndrome, with the rate of publication increasing over recent years. The largest case series now includes 152 individuals, almost all heterozygous for one or both of the two mentioned variants. However, to date, there is insufficient evidence to link the broad phenotypes described in Yao syndrome to variants in *NOD2*. Approximately one in five people is heterozygous for c.2717+158C>T in the gnomAD database, while one in 12 is heterozygous for p.Arg702Trp in gnomAD. Thus, while it is conceivable that there is an association between very common variants in *NOD2* and the phenotypes referred to as Yao syndrome, this could only represent susceptibility with extremely low penetrance. Demonstrating such a link would require large-scale association studies, which have not been performed. As a result, given the current lack of evidence for the existence of Yao syndrome, this diagnosis should not be used.

## Teaser

There are 30+ papers published on NOD2-autoinflammatory (Yao) syndrome. However, approximately one in five people is heterozygous for Yao-associated variants, so any relationship between variants and clinical phenotypes can only represent extremely low penetrance. Large association studies are needed to prove an association, which have not been done, and the diagnosis should not be used.

Methodologies that define variant-disease or gene-disease associations differ depending on the strength of that association. Weak associations, in which the variant contributes a small percentage of the genetic predisposition, require the statistical power of large association studies (typically hundreds or thousands of individuals) to confirm the association. On the other hand, association studies may not be required to define variant-disease associations in which the variant is responsible for all or most of the disease phenotype—such as monogenic inborn errors of immunity (IEIs). In such instances, a powerful case may be constructed using multiple differing but intersecting pieces of evidence, each of which supports a gene-disease association. However, this relies on adherence to very specific rules for attribution of pathogenicity. To avoid misattribution of pathogenicity, one of the cardinal rules for defining a new IEI is that the frequency of the candidate genotype should not be greater than the disease prevalence, allowing for penetrance (1).

NOD2-associated autoinflammatory syndrome, or Yao syndrome, was first put forward as a potential autoinflammatory syndrome associated with *NOD2* variants in 2011, with almost all cases to date associated with two variants originally reported as IVS8+158 (MANE transcript NM_001370466.1: c.2717+158C>T/rs5743289) and R702W (Matched Annotation for NCBI and EBI [MANE] transcript NM_001370466.1: c.2023C>T;p.Arg675Trp/rs2066844) (2). The clinical description of Yao syndrome includes periodic fever, dermatitis, polyarthritis, serositis, and negative serum autoantibodies, and the authors of the initial paper presented *NOD2* variants as being required for the diagnosis, essentially defining this as an IEI associated with *NOD2*. Following that initial report, three subsequent reports came out from the same team across the 2010s (3, 4, 5), with the first report from an alternative source coming in 2018 (6). There are now ∼35 publications mentioning Yao syndrome, roughly half of which include Dr. Yao as an author (2, 3, 4, 5, 7, 8, 9, 10, 11, 12, 13, 14, 15, 16). Reports of individuals with Yao syndrome have increased in recent years, with one paper including Yao syndrome published in 2020 (17); two in 2021 (12, 18); five in 2022 (8, 14, 15, 19, 20); three in 2023 (9, 11, 21); eight in 2024, including two case series (10, 13, 22, 23, 24, 25, 26, 27); and four already in 2025 (24, 26, 28, 29). A number of papers now advise specific therapies for this entity, including complex biologics (9, 19, 25, 29).

However, notwithstanding this extensive literature, the available evidence is not sufficient to support the existence of Yao syndrome as a clinical entity.

Firstly, the two variants reported in most individuals with Yao syndrome are vastly too common to represent variants with a Mendelian effect, and submitters to the ClinVar database have classified both c.2717+158C>T and p.Arg675Trp as either variant of uncertain significance, likely benign, or benign (variation IDs: 4697 and 4693, respectively). This fact was acknowledged in a recent publication (Nomani and colleagues), which accepted that population data do not support a monogenic disease model for Yao syndrome (10).

On the other hand, *NOD2* variants could contribute to the phenotypes ascribed to Yao syndrome in a non-Mendelian manner by participating in polygenic inheritance. However, large association studies would be required to demonstrate such a role, and these have not been performed.

Another way of connecting the genotype to a putative rare disease phenotype is that they could represent hypomorphic alleles only associated with disease when potentiating the effects of a powerful rare variant. In such a case, the rarity of the rare variant accounts for the rarity of the disease (e.g., common variants potentiate rare deletion alleles in thrombocytopenia absent radius syndrome [30]). However, exome or genome sequencing studies of cohorts of patients with Yao syndrome have not been conducted, and additional rare variants have not been identified in this entity.

The paper by Nomani et al. did propose an alternative mechanism, termed “genetically transitional disease” in which genetic variants are necessary but not sufficient to cause disease. This concept essentially equates to the phenomenon of incomplete—and low—penetrance. Well-studied examples of this include hemochromatosis, Leber hereditary optic neuropathy, and *DYT1*-associated dystonia. However, there is not currently evidence to support such a genetic mechanism for Yao syndrome. One study did attempt cohort analysis from 143 autoinflammatory cases but with a number of technical issues. Inclusion criteria were vague (periodic disease consisting of fever, rash, and arthralgia, among other signs and symptoms); the control data were selected from historical literature, and the authors did not correct for multiple comparisons (5). Even with these limitations, the lower limit of the confidence interval for the odds ratio was zero, consistent with there being no effect at all. At best, the data support only a very modest risk for disease and do not support a causative genetic diagnosis.

It seems unlikely that evidence for a low-penetrance relationship between common alleles in *NOD2* and Yao syndrome will ever be identified. This is because a central requirement for identifying alleles with a weak association to disease is the preexistence of a defined clinical phenotype in cohorts of patients, where that genetic association can be tested. The clinical phenotype described as Yao syndrome did not exist in the literature prior to the initial description of an association with variants in *NOD2*. Given the common nature of features such as rash and arthralgia, there is still no clearly delineated clinical entity that could be used to confidently define cases in an association study.

The question then is, if there is no such thing as clinical Yao syndrome, how did the entity arise? In 2011 when Yao syndrome was first described, variants in *NOD2* had been generating significant excitement as Crohn's disease susceptibility alleles (31), including c.2717+158C>T (32) and p.Arg675Trp (33). Large population databases of genetic variation were not yet available, and when they did come online, they included mostly exome data, without information about the frequency of deep intronic variants. As a result, the c.2717+158C>T variant did not appear in large population databases until the launch of gnomAD v3, in 2019, although it was present in HapMap (rs5743289). Additional factors may have contributed to erroneously drive the association of common variants to common clinical features. First, there is a low rate of monogenic autoimmune and autoinflammatory disease among adult patients, meaning there is a low pretest probability of reaching a true single gene diagnosis in this patient cohort (34). Second, some commercial genomic sequencing providers report extremely common Inflammatory Bowel disease (IBD)-risk alleles in *NOD2* on immune panel genomic testing, increasing the chance that these variants may be attributed by non-geneticist clinicians as underlying a monogenic diagnosis. In this context, it seems likely that clinicians desiring to provide a diagnosis for adults with autoinflammation may have overinterpreted the clinical panel results, labeling patients as having Yao syndrome. Given the frequency of these variants in normal populations, it is perhaps not surprising that a cohort of nearly 200 cases was generated (10).

In summary, the variants in *NOD2* that are reported as causative of the vast majority of Yao syndrome are too common to be Mendelian disease associated. Those variants have not yet been studied in large cohorts to establish a statistical association to a distinct phenotype or to demonstrate that they represent susceptibility alleles. Such cohort studies would be challenging, perhaps impossible, to conduct, because Yao syndrome is not an entity diagnosable independently of the proposed association with *NOD2*. Taking this information into consideration, our view is that Yao syndrome does not exist, and unless and until further evidence is published that addresses these issues, the term should no longer be used in the medical literature, or in clinical practice.
